# A Treatise on Strabismus or Squinting, and the New Mode of Treatment, Illustrated by Engravings and Cases

**Published:** 1841-07-10

**Authors:** 


### MEDICAL EXAMINER.

DEVOTED TO MEDICINE, SURGERY, AND THE COLLATERAL SCIENCES.

No. 28.] PHILADELPHIA, SATURDAY, JULY 10, 1841. [Vol. IV.

### BIBLIOGRAPHICAL NOTICE.

*A Treatise on Strabismus or Squinting, and the new mode of Treatment, illustrated by Engravings and Cases.* By John H. Dix, M. D., member of the Massachusetts Medical Society.

We have received this neat little work, and would cheerfully give it a more extended notice, if we had not already worn threadbare the subject of Strabismus. We recommend it, however, to those of our brethren to whom the new mode of treatment may be still a novelty. There are doubtless many physicians residing in the interior, whom it has reached only as a rumor, who have had no opportunity of witnessing the new mode of treatment, or learning its results. To such the little volume of Dr. Dix would be extremely serviceable. It is true that it does not give the various modifications by which the operation has been rendered more prompt and less painful, but it embodies all that is essentially necessary to its performance. A chapter upon the anatomy and functions of the muscles of the globe, chiefly extracted from Sir Charles Bell, is followed by a clear description of the operation as practised by Dr. Dix, accompanied by an illustrative plate, while the remainder of the work consists of the details of twenty-seven cases in which this method has proved successful, and concludes with the following

ANALYSIS OF FIFTY CASES.

| | | |
|---|---|---|
| Females, | 31 | |
| Males, | 19 | —50 |
| Convergent, | 48 | |
| Divergent, | 2 | —50 |
| One eye only squinted and was operated on in | 36 | |
| Double,—that is, requiring the operation on both eyes, in | 14 | —50 |

Three cases are supposed to have been congenital, and the squint was observed previous to the eighth year in every case but two.

The causes assigned were as follows: Fits, 6. Inflammation, 5. Hooping cough, 5. Chorea, 1. Blow, 2. Fracture of skull, 1. Imitation, 11. Unknown, 19.

With regard to the results of the operation, Dr. Dix thinks that no reasonable doubt can be entertained of the permanence of the cure by this mode of treatment.

"It is hardly possible that a recurrence of the remote causes, which is in adult life an exceedingly unlikely event, should produce the same effect as before; for in most cases it may be presumed, that immediately after the division, if not before, the muscle attains nearly the utmost contraction of which it is capable, and the distance from its origin to its insertion being less, the same increase of morbid action as before could not, to the same extent, change the position of the globe. In my earliest cases, which were operated on not quite six months ago, no tendency to relapse has been exhibited, but, on the contrary, in some of them a more complete assimilation of the motions of the eyes has taken place in the course of this time. Sixteen months have elapsed since Professor Dieffenbach first operated in Europe, and in a report of his cases no allusion is made to a return of the complaint in any instance.

—

The present is not an unfavorable opportunity to connect with the report of Dr. Dix the results of cases published by other operators. The number of cases now recorded is sufficiently great to render their analysis both interesting and instructive, and a sufficient length of time has elapsed since the first introduction of this operation, to justify an opinion upon its probable permanent effects. We may premise that Dr. Forbes has learned from a German Journal that the operation in question was performed once at Dresden, previously to its performance by Dieffenbach at Berlin. Stromeyer has undoubtedly the credit of having first suggested it, and as it has been destined to make some noise in the surgical world, the present appears the proper time to settle the question of priority in regard to its performance.

We are indebted to the British and Foreign Review for the great mass of materials from which we have deduced the following statistics. To the cases of Melchior, Lucas, Duffin, Amnor, Colder, Phillips, &c. to be found in that Journal, we add those reported in detail in this country, and find that out of 536 cases of strabismus, reported by different persons, 506 were convergent, and of these 459 affected one eye only, and in 47 the convergence existed in both eyes. Divergent strabismus is much less frequent; out of 866 cases it only presented itself 44 times, and M. Lucas states that he has only seen it once in some hundreds of cases of squint which he has examined. The statistics in regard to the relative frequency of distortion of the eye, upwards or downwards, are not so full. Out of 161 cases reported by Phillips and Melchior, there were only 4 upwards and 2 downwards. The distortion, however, is not confined to the line of action of a single straight muscle; it may occur in a line between any two. The number of cases in which this species of obliquity has been noticed is very limited. In the paper of Dr. Pancoast, in No. 25 of the Examiner of the present year, it will be found that out of 55 cases "in 15 the pupil was directed upwards and inwards, or took this position habitually when the patient was agitated from any cause; in five, the squinting was obliquely inwards and downwards." It appears, also, that the left eye is more disposed to squint than the right; thus, out of 386 cases, the left eye was exclusively affected 204 times and the right eye 119 times, and M. Lucas marks the proportion in favor of the left eye as 3 to 2.

This preference for the left side is interesting, and may tend to throw some light upon the cause of the deformity—to decide whether it depends upon active and permanent muscular contraction, or upon a disparity in the strength of the two organs,—upon a comparatively defective vision of one eye. The predominance of certain diseases in the right or left side of the body, is so uniform, that it must be referred to some fixed cause; and this cause is not irrationally explained by the theory of Serres, in relation to the development of the fœtus; formed of two lateral halves, one side is almost always more developed than the other, and the greater vigour of this side is natural, not acquired; and is permanent.—Ordinarily, it is the right side which is most developed, and hence the majority of individuals are naturally right-handed. Occasionally, the predominance is on the left side, and a naturally left-handed person is born; and in cases still less frequent, both sides have an equal development, and a natural ambidexter is the consequence. The disproportion in the vigour of the two sides, does not then depend upon the unequal exercise to which they are subjected, although instinct induces us to rely more upon the one most developed, and thus tends still further to augment its development; but the difference existed *ab initio*. But again, the disproportion of the two sides is not confined to the extremities, but will be found to exist to a proportional extent between the two halves of every symmetrical organ, and to extend to all the organs of the life of relation, the eyes included: and as a corollary, may be supposed to exercise an influence on the character of the diseases to which either side is most liable—thus, *à priori*, we would be led to seek for those affections which are the result of over-action, upon the right side of the body; (we speak of right-handed individuals;) while congenital deformities, the result of an arrest of development during uterine life, and diseases dependent upon a want of action after birth, would be sought for upon the left side. What theory would suggest, observation confirms.

Those diseases dependent upon undue vitality, as the formation of supernumerary organs &c., will be found on the right side—while those which betray a want of energy, as partial or total atrophies, &c., will be met with more frequently on the left.

As we are noticing the opinions of others, rather than advancing our own, we are indisposed at present to frame an explanation of the greater frequency of strabismus on the left side, from the laws which we have just announced, and which are almost demonstrable. We content ourselves with offering them as a suggestion to those who make this subject their more especial study. We cannot however omit laying before our readers, from the British and Foreign Review, the following communication, which has a strong bearing upon the point. It was recently made verbally to the Royal Academy of Medicine of Paris, by M. Bouvier:

A woman eighty-two years of age, having died at the Salpêtrière, with divergent strabismus of the left eye, with which, according to her own report she had been affected from infancy, M. Bouvier siezed the opportuniiy to examine what changes the muscles of the eye experience in strabismus.

"I remarked," he says, "first that the eye, which still remained everted, could be easily pushed inwards. To this the external straight muscle offered not the least resistance, although during life when the sound eye was closed, the patient could turn the eye from without inwards no farther than to the middle of the orbit. The dissection of the muscles which I present to rhe Academy, demonstrates that the external ectus is in a state of complete relaxation, and that its length is sensibly the same as that of the other straight mnscles, so that the eye can be easily moved in all directions. Nor is the texture of the external rectus altered in any way."

M. Bouvier's reflections on this case are so just, that we do not hesitate to quote them at length:

"If, as may be supposed, the disposition which existed in this case is general, it would follow that strabismus would not be owing, like club-foot, to a permanent muscular contraction. It would depend solely on the physiological action of, or a habit of contraction in certain muscles, an opinion which is confirmed by the instantaneous return of the eye to its proper situation and movements, in most cases, as soon as the sound eye is covered. From this it would result that, although it was the success of tenotomy in the contractions of limbs which led M. Stromeyer to propose, and Dieffenbach to perform the section of muscles of the eye in strabismus, there is no analogy in the two cases, since the effect of the operation in strabismus would not consist simply in the destruction of a physical resistance, but rather in the modification of a physiological action, and in the establishment of harmony in the muscular contraction of the right and left sides. Such would be the condition of the success of the operation, if this be confirmed by time." (Bull. d l'Acad. de Méd.)

The most valuable information we possess, however, on the state of the muscles of a squinting eye, have been derived, not from *post-mortem* examination, but from observations made during operation. "In the operation for squinting," says Mr. Guthrie, "the muscle usually divided does not appear to be in the least diseased, but on the contrary, quite in its natural state." In many instances an hypertrophied state of the muscle is reported to have been met with. Thus, Mr. Lucas tells us (p. 28,) he has "had many opportunities of witnessing the great development of the inner rectus muscle in convergent strabismus. In such cases the muscle was not merely increased in bulk, but was also much more vascular, and of a deeper colour than natural—conditions which remarkably contrast with the appearance the muscles of the eye present in their healthy state."

Is such hypertrophy of the muscle, cause or effect?

Dr. Von Ammon also, although he has always noticed in cases of convergent strabismus, a contraction, a remarkably blanched appearance, not unfrequently a dryness, and an abnormal density and thickness of the "*conjunctiva bulbi*," concludes:

As to the morbid condition of the muscles in strabismus—when the inversion was very great, the insertion of the internal rectus was often found farther back than usual. I have observed the same thing in divergent strabismus; in the majority of cases, however, the insertion of the muscle was normal. As regards the muscular substance itself, it appeared to me sometimes thick, gorged with blood, pouring out after division a considerable quantity of blood, and less easy to divide than a sound muscle: in such cases it was rather round than flat. Sometimes it appeared as if the muscle was very tendinous, and then either very thin and shrunk, or tough and pretty thick; it had in that case lost the peculiar muscularity almost entirely, so that the division of it was attended with a creaking sound. *Very often, however, I have observed nothing abnormal in the muscle, the subject of operation, whether in colour, consistence, or length.*

The exciting causes of strabismus are well worthy of study, in reference to its prophylaxis. This deformity is rarely congenital, but is frequently the effect of voluntary acts on the part of the individual; and might therefore have been avoided, had their danger been known. Thus, out of 321 cases in which the exciting causes were investigated, we are able to make the following distribution:

| | |
|---|---|
| Imitation, | 61 |
| Looking fixedly or suddenly at o bjects in youth, | 16 |
| Looking at an object on the eyebrow, nose, cheek, inner canthus, thumb, while held in the mouth, | 9 |
| Holding the head sideways while knitting, | 3 |
| Watching the motion of a shuttle, | 1 |
| Looking at Sun, | 2 |
| Exposure in infancy to light and heat of fire, | 3 |
| Fit of crying, | 1 |
| Injuries inflicted on the eye, | 15 |
| Severe whipping, | 1 |
| Excessive fright, | 4 |
| Falls on head, | 8 |
| Injury of head, | 2 |
| From falls, | 5 |
| From blows, | 2 |
| Concussion of brain, | 1 |
| Fracture of skull, | 1 |
| Convulsions, | 31 |
| Epilepsy, | 2 |
| Chorea, | 1 |
| Difficult dentition, | 8 |
| Intestinal worms, | 3 |
| Hooping cough, | 11 |
| Measles, | 14 |
| Small-pox, | 11 |
| Opthalmia, | 36 |
| Inflammation, | 5 |
| Imperfect cataract, | 3 |

| | |
|---|---|
| Amaurosis, | 2 |
| Ill health, | 1 |
| Burns of abdomen, | 2 |
| Supposed congenital, | 17 |
| Unknown, | 40 |

Thus we see that imitation alone has been productive of more cases of strabismus than any one other cause—while, if we take into consideration the number of cases the result of imprudence on the part of the individual, or of others, we shall find that they amount to 116: while those the result of disease, or supposed congenital, do not exceed 164. What a caution is contained in this fact!

